# Measuring Air Quality for Advocacy in Africa (MA3): Feasibility and Practicality of Longitudinal Ambient PM_2.5_ Measurement Using Low-Cost Sensors

**DOI:** 10.3390/ijerph17197243

**Published:** 2020-10-03

**Authors:** Babatunde I. Awokola, Gabriel Okello, Kevin J. Mortimer, Christopher P. Jewell, Annette Erhart, Sean Semple

**Affiliations:** 1 Centre for Health Informatics, Computing & Statistics (CHICAS), Lancaster Medical School, Lancaster University, Lancaster LA1 4YW, UK; c.jewell@lancaster.ac.uk; 2 Department of Clinical Sciences, Liverpool School of Tropical Medicine, Liverpool L3 5QA, UK; Kevin.Mortimer@lstmed.ac.uk; 3 Department of Clinical Services, Medical Research Council Gambia at London School of Hygiene & Tropical Medicine, P.O. Box 273 Banjul, Gambia; 4 Institute for Sustainability Leadership, University of Cambridge, 2 Trumpington Street, Cambridge CB2 1QA, UK; g.okello@africancentreforcleanair.org; 5 African Centre for Clean Air, P.O. Box 4357 Kampala, Uganda; 6 Respiratory Medicine Department, Aintree University Hospital NHS Foundation Trust, Liverpool L9 7AL, UK; 7 Disease Control & Elimination Theme, Medical Research Council Gambia at London School of Hygiene & Tropical Medicine, P.O. Box 273 Banjul, Gambia; aerhart@mrc.gm; 8 Institute for Social Marketing and Health, University of Stirling, Stirling FK9 4LA, UK; sean.semple@stir.ac.uk

**Keywords:** PM_2.5_ monitor, ambient air pollution, measurement sensor, low-cost, feasibility, sub-Saharan Africa

## Abstract

Ambient air pollution in urban cities in sub-Saharan Africa (SSA) is an important public health problem with models and limited monitoring data indicating high concentrations of pollutants such as fine particulate matter (PM_2.5_). On most global air quality index maps, however, information about ambient pollution from SSA is scarce. We evaluated the feasibility and practicality of longitudinal measurements of ambient PM_2.5_ using low-cost air quality sensors (Purple Air-II-SD) across thirteen locations in seven countries in SSA. Devices were used to gather data over a 30-day period with the aim of assessing the efficiency of its data recovery rate and identifying challenges experienced by users in each location. The median data recovery rate was 94% (range: 72% to 100%). The mean 24 h concentration measured across all sites was 38 µg/m^3^ with the highest PM_2.5_ period average concentration of 91 µg/m^3^ measured in Kampala, Uganda and lowest concentrations of 15 µg/m^3^ measured in Faraja, The Gambia. Kampala in Uganda and Nnewi in Nigeria recorded the longest periods with concentrations >250 µg/m^3^. Power outages, SD memory card issues, internet connectivity problems and device safety concerns were important challenges experienced when using Purple Air-II-SD sensors. Despite some operational challenges, this study demonstrated that it is reasonably practicable and feasible to establish a network of low-cost devices to provide data on local PM_2.5_ concentrations in SSA countries. Such data are crucially needed to raise public, societal and policymaker awareness about air pollution across SSA.

## 1. Introduction

Exposure to ambient air pollution is increasingly recognized as a serious threat to human health [1]. The Lancet commission on air pollution in 2017 suggested that approximately 92% of pollution-related deaths occur in low-and-middle-income countries (LMICs) [2]. However, the magnitude of the risk attributable to ambient air pollution is largely unknown in sub-Saharan Africa (SSA) and largely extrapolated from data obtained from LMICs outside SSA or from household air pollution studies in SSA. Exposure to air pollution is associated with a wide range of diseases including Chronic Obstructive Pulmonary Disease (COPD), asthma, lung cancer, heart disease, stroke, arterial thrombosis and hypertension [1,3,4,5,6].

High-income countries (HICs) have seen major improvements in health impacts attributable to reduced ambient air pollution over recent decades. These health impacts include reduced risks of premature death associated with exposure to ambient fine particle pollution [7,8], reduced risks of premature mortality [9], reduced number of people with illnesses [10,11], reduced number of emergency visits; and reduced number of lost school- and work days [9]. These recent achievements are mainly due to the increasing body of evidence on air quality in indoor and outdoor spaces and increasing public awareness and advocacy on the health impacts of air pollutants. These were supported by the development of rigorous evidence-based national and supranational public health policies, such as Clean Air Act [9] and Ambient Air Quality Directives [12], as well as locally based interventions like creating Lower Emission Zones in cities.

The collection of air quality data and increase of awareness have been bolstered by the proliferation of low-cost, user-friendly air pollution online platforms (e.g., Air Apparent, Bristol, UK; Love Lambeth Air, London, UK; Luftdaten, Germany and Europe, etc.) involving the general public in measuring and monitoring air quality at high spatial resolution and nearly in real time [13,14,15]. These platforms enabled a wide range of initiatives, i.e., from correlation studies of low-cost particulate matter (PM_2.5_) monitors which have been used to compare and calibrate low-cost devices against gravimetric or reference monitors [16,17,18] to citizen science projects focused on community behavioral change such as Friends of the Earth’s “Clean Air Campaign”; Spectropolarimeter for Planetary Exploration- iSPEX Netherlands and Europe [19,20]. The air quality information, including forecasting of air pollution levels, has been disseminated to citizens through various channels such as websites (e.g., AirVisual App by IQAir AG, Zurich, Switzerland), newspapers and text messages (e.g., *air*TEXT from Cambridge Environmental Research Consultants, Islington, UK) and phone applications (e.g., AirLief App from Smart Fab Lab, Sofia, Bulgaria) [21,22,23,24].

Combating air pollution is a low priority on the public health and policy agenda of many governments in SSA which helps to explain the limited availability of routinely collected ambient air pollution data (PM_2.5_ data) (Supplementary file 1) and absence of national policies in most countries [25]. However, this situation could be rapidly improved considering the advent of highly portable, yet efficient, air quality monitoring (AQM) instruments. As in HICs, these tools are likely to make air quality measurement affordable and accessible to scientists and non-scientists alike, making citizen science a reality in SSA [26]. Indeed, studies comparing the performance of newer less expensive AQM instruments to the more expensive gold standard instruments have revealed very promising results [27,28,29,30]. The performance of the GRIMM reference method versus Purple Air sensor (Purple Air, UT, USA) revealed a good level of agreement with *R*^2^ value of 0.98 [3,31] (Supplementary file 2). These independent evaluation data from Air Quality Sensor Performance Evaluation Centre (AQSPEC) field evaluation report which revealed a *R*^2^ of 0.98 as stated above were free of commercial and promotional influence [31]. It is worth noting that much as the Purple Air monitors seem to perform well, there is a wide array of low-cost sensors and the different low-cost sensors have reported varying levels of precision/accuracy in different contexts [17,27]. It is important to establish the setting of the environment when considering the type of low-cost sensors to be deployed. The conditions such as availability of electricity for powering sensors, internet connection (to upload data online in near real time), safety of the devices, weather conditions (humidity or extreme weather) are important considerations for sensor deployment. Applying these low-cost technologies offers a potential opportunity for long-term exposure measurements and determination of drivers/sources of air pollution in SSA.

We hereby present the results of an observational multi-country study evaluating the feasibility and practicality of longitudinal ambient PM_2.5_ measurement using low-cost sensors in 13 locations across sub-Saharan Africa. The focus of this work was not determining the accuracy or precision of the measurements in terms of instrument calibration, but rather on assessing the feasibility and practicality of installing and using these devices in typical low-and-middle-income (LMIC) settings. There is a need to understand the barriers to collecting air quality data in SSA settings and this manuscript sets out to address that knowledge gap. We believe that providing the research community with information on how to develop these methods and identifying potential barriers to data collection is critical to air pollution data collection in settings like SSA. Furthermore, our research team has a stepwise approach to addressing the issue of ambient air pollution in SSA, with the first stage described in this manuscript being piloting low-cost sensors to provide real-time and widely available data. The second stage will be to gather data over a full 12-month period from across our network of monitors, with the third stage involving use of these data to generate advocacy and policy discussions with relevant local, national and regional stakeholders. Please note that instrument calibration and the validity of measurements from the Purple Air sensors have been previously described by the independent Air Quality Sensor Performance Evaluation Centre [3,31].

## 2. Materials and Methods

### 2.1. Study Design and Study Sites

This was a collaborative research project between Lancaster University, the Liverpool School of Tropical Medicine and the Measuring Air Quality for Advocacy in Africa (MA3) initiative of the African Centre for Clean Air (ACCA). The study involved four weeks of pilot longitudinal air pollution monitoring carried out across 13 locations in seven SSA countries.

**Figure 1:**
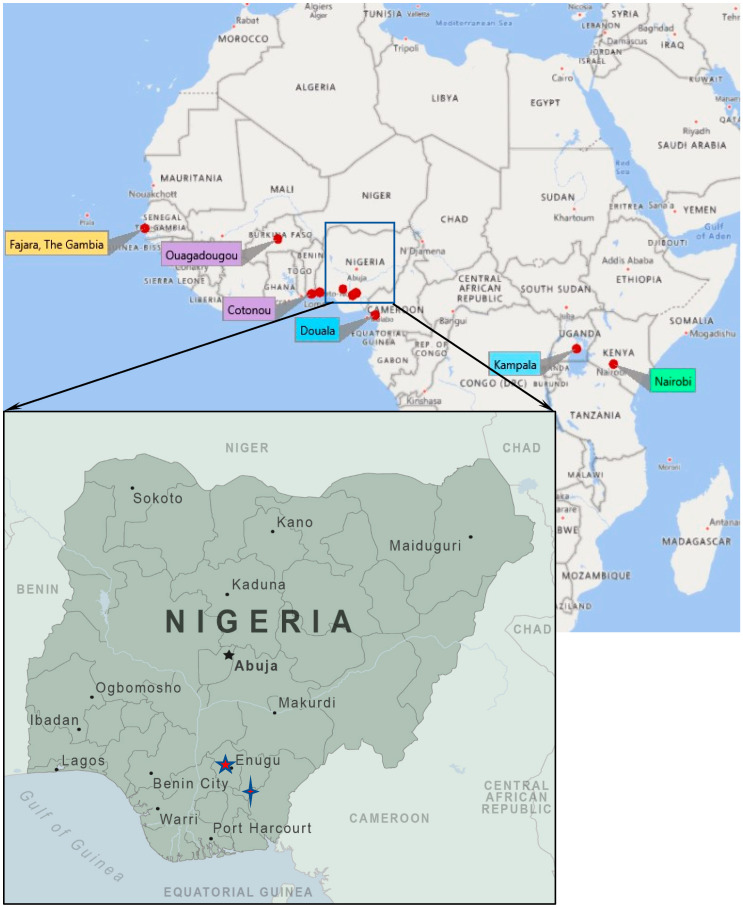
Map of sub-Saharan Africa showing the 13 study sites (including 4 in Enugu, and 2 in Anambra, both in Nigeria) across 7 countries. 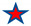
—Four sites in Enugu, Nigeria, 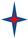
—Two sites in Anambra, Nigeria (Awka and Nnewi).

Data on ambient PM_2.5_ were collected continuously from 1st to 31st July 2019. This was intended to be a pilot study in preparation for a one-year longitudinal PM 2.5 measurement in the following 13 sites (Figure 1 below):

1. Cotonou, Benin Republic
2. Ouagadougou, Burkina Faso
3. Douala, Cameroon
4. Fajara, The Gambia
5. Nairobi, Kenya
6. Bariga, Lagos, South-Western Nigeria
7. New Haven, Enugu, Eastern Nigeria
8. Goshen, Enugu, Eastern Nigeria
9. Abakaliki Road, Enugu, Nigeria
10. Trans-Ekulu, Enugu, Eastern Nigeria
11. Awka, Anambra, Nigeria
12. Nnewi, Anambra, Nigeria and
13. Kampala, Uganda [3]

### 2.2. Recruitment of Participants

Study sites were chosen among the home institutions of the clinical and basic science researchers that participated in the International Multidisciplinary Programme Against Lung Diseases and Tuberculosis in Africa (IMPALA)/Pan African Thoracic Society Methods in Epidemiologic, Clinical and Operations Research (PATS MECOR) course held in June 2019 [3,30,32]. During this course, each participant was given one Purple Air II SD device and one 20,000 mAh long-lasting portable Anker^®^ power bank (Anker Innovations, Changsha, China).

### 2.3. Data Collection

#### 2.3.1. Device Setup

The standard operating procedures for assembling, connecting and putting up the Purple Air-II-SD device were taught at the above-mentioned workshop, including practical hands-on sessions. Selection criteria for the Purple Air-II-SD device mounting point in each site were the following: (i) the device is sited away from obstructions such as trees and fences and at a reasonable distance from the source of ground dust like unpaved road, rooftop air inlet; (ii) the device is at a good distance away from a road with heavy traffic, i.e., minimum 100 m from dusty or heavily plied roads; (iii) the entire device is placed at two meters from the ground level for uniformity and ease of data comparability; (iv) the device is sited away from grills, generators, incinerators, air conditioning vents and any other non-traffic particulate matter source.

#### 2.3.2. Measurement of PM_2.5_

Measurements of ambient PM_2.5_ concentration were carried out using Purple Air-II-SD devices with firmware versions 3 (not connected) and 4.02 (connected to Wi-Fi) [33]. The devices logged PM_2.5_ concentration at intervals of 80 s for firmware version 3.0, and of 120 s for firmware version 4.02. The devices were connected to Anker Pro Power banks in areas where power supply was unreliable or was difficult to access.

#### 2.3.3. Data Sampling

Baseline data about the sampling site was collected using a standard pre-coded questionnaire (Supplementary file 1). Information regarding the challenges faced during installation, use, maintenance, and data download from the PurpleAir-II-SD device was collected continuously during the 31-day follow-up using a separate monitoring form (Supplementary file 3). The 24 h PM_2.5_ concentration was calculated daily from the collected data. In sites where devices were not connected to Wi-Fi, Microsoft^®^ Excel (Microsoft^®^, Redmond, WA, USA) Comma Separated Value (CSV) files containing the ambient PM_2.5_ data were downloaded manually every week by the site coordinator and sent by email to the lead researchers [3]. In sites where the device was connected to wifi, the PM_2.5_ data were uploaded continuously onto the Purple Air website [26], from where they were extracted and analyzed.

The Purple Air-II-SD has an in-built Real-Time Clock (RTC) that sets itself when connected to the internet. Tests were carried out to quantify the degree of drift when the device was used in SD-logging mode (i.e., not connected to the internet) for extended periods [3].

### 2.4. Data Analysis

PM_2.5_ data were received from all the participating sites for a period of four weeks. Each of the seven CSV files (one per day) contained PM_2.5_ data logged at either 80 s (version 3.0) or 120 s intervals (version 4.02). Each file was screened and cleaned for errors before being run through an in-house, bespoke software to extract daily PM_2.5_ averages by date and create a single CSV file per study site.

The data recovery rate was calculated as the percentage number of hours for which PM_2.5_ data were logged during the study period divided by the maximum potential number of hours based on the device sampling rate. Summary statistics were used to compute average values of daily, hourly and period PM_2.5_ concentrations, as well as the frequency distribution of measured values by PM_2.5_ threshold categories using the Microsoft Excel^®^ (Microsoft^®^, Redmond, WA, USA) 365 Pro-plus environment [3].

Information regarding challenges faced by the exposure scientists at all sites was documented by individual scientists and sent via email to the principal investigator. This was designed as a self-reported list of challenges encountered during the research project. Specifically, challenges around device setup, device maintenance (powering, connecting to Wi-Fi), data download and any other important issues were requested.

### 2.5. Ethical Permission

Ethical waiver was given for the study by the Research Ethics Committee the Liverpool School of Tropical Medicine (LSTM REC number 19-061) considering the absence of human or animal subjects.

## 3. Results

### 3.1. Baseline Characteristics of Study Sites

Of the seventeen clinical and basic science researchers who participated in the AQM workshop [30], thirteen representing seven SSA countries participated and contributed data to the study (Table 1). Four researchers (from Kenya, Tanzania and Sudan) withdrew from participation for personal reasons. Twelve sites were urban, and one was semi-urban in Enugu, Nigeria. Devices were mounted mainly in residential areas except in three sites where they were in hospital premises, i.e., Cotonou (Benin), Douala (Cameroon) and Lagos (Nigeria). Data collection was carried out in July: a time that generally corresponded to the rainy and wet season in all sites.

Out of the thirteen Purple Air-II-SD study devices, only two, namely, Nairobi (Kenya) and Fajara (The Gambia), were permanently connected to the cloud via Wi-Fi; thus data were downloaded manually every week in the other eleven sites.

### 3.2. Data Recovery and PM_2.5_ Concentration Measurements per Site

In one site (Douala (Cameroon)), the device failed to log any data due to technical difficulties. The other 12 sites achieved >90% data recovery (median 94.7%, IQR (93.2; 97.1), except Lagos (Nigeria) where the recovery rate was only 72.1% due to data loss following the wrong placement of an SD memory card for a period of sampling (Table 2). In Goshen (Nigeria), the device logged data more frequently than expected due to a firmware problem that caused it to search for Wi-Fi to re-set the time on the real-time clock. This led to an excess of logged records (105.5% of anticipated) at this location.

Furthermore, Table 2 shows the distribution of all period PM_2.5_ measurements by categories defined by increasing thresholds, i.e., >10 μg/m^3^, >25 μg/m^3^ and >250 μg/m^3^; the World Health Organization (WHO) recommended value for daily average being ≤25 μg/m^3^. In half of the 12 sites, 60% or more of the measured values were above the WHO threshold—with rare records of >250 μg/m^3^, Lagos (Nigeria) and Kampala (Uganda) being the most polluted with, respectively, 96% and 92% of values being >25 μg/m^3^. Among the other six sites, the majority of measurements were below the WHO threshold, with The Gambia site (Fajara) ranking as the least polluted (44.5% of measurements ≤10 μg/m^3^).

### 3.3. Comparison of Daily Average PM_2.5_ Concentrations against WHO PM_2.5_ Recommended Threshold (25 μg/m^3^)

**Figure 2:**
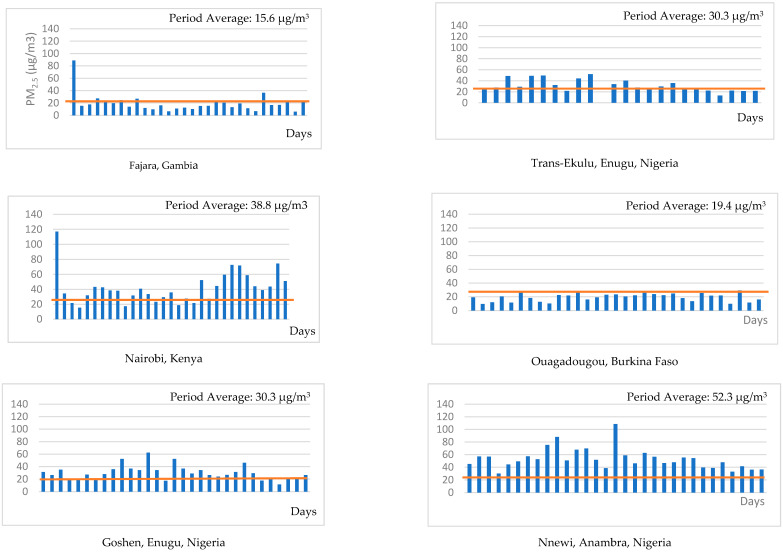

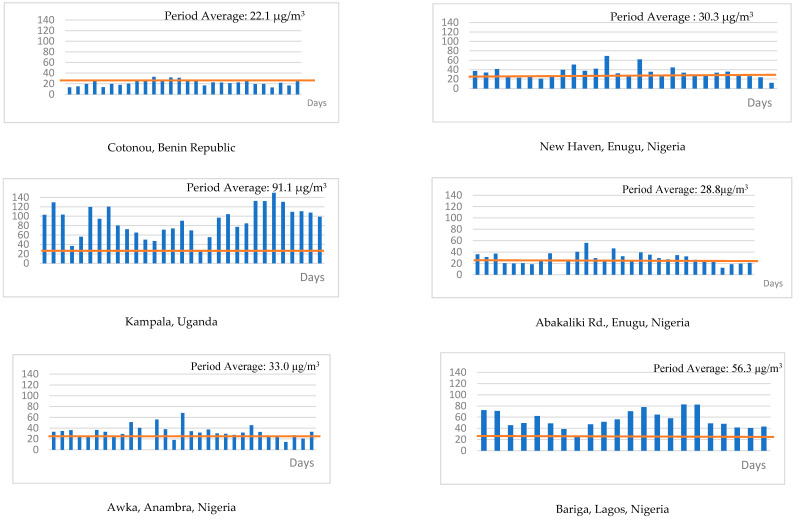
Daily average PM_2.5_ concentrations by site measured from 1st to 31st July 2019 in the MA3 pilot study. Y-axis represent PM_2.5_ concentrations in μg/m^3^.

Figure 2 shows the daily PM_2.5_ concentration in each of the study sites with a red line showing the WHO PM_2.5_ recommended threshold for daily levels (25 μg/m^3^). Kampala, Nairobi, Lagos and Nnewi in Nigeria showed daily values that were well above the WHO threshold. On the other hand, the Fajara, Gambia and Ouagadougou, Burkina Faso sites had daily averages well below the WHO daily threshold of 25 μg/m^3^. Others had borderline values, very close to the WHO threshold, such as Cotonou, as well as the four Enugu sites and Awka in Nigeria [3].

### 3.4. Challenges Identified While Using the Purple Air-II-SD AQM Sensors

#### 3.4.1. Real-Time Clock Stability

The time drift experienced by the devices was calculated in twelve logging sites during the final week by each investigator (see protocol in Supplementary file 4). Cameroon was excluded because the firmware of the device was corrupt. The RTCs showed a ‘drift’ of 4 to 23 min over the period of use, suggesting that the clocks may lose between about 8–10 s to nearly 1 min per day if not connected to the internet to ensure they continue to log data at the correct time.

#### 3.4.2. Practical Challenges Encountered by Exposure Scientists at all Measurement Sites

Table 3 summarizes the results of the survey on challenges identified by study participants in relation to the installation and use of the Purple Air device during the MA3 study. Power outages and related costs was the most frequent challenge reported by 7 out of 12 investigators. Specifically, one user had to buy a second power bank, given the long charging time, and two users reported the need for electricity generators. By ceasing every available opportunity to ensure continuous powering of the devices, the others checked the device power source often and made sure the power pack was fully charged. The second most important challenge was device setup issues as reported by half of the users. These included mainly finding a suitable and safe location to mount the device (*n* = 2) and incurring extra costs for assisted device setup (*n* = 2); only one user reported a Wi-Fi connection problem. One investigator reported difficulties in finding a location that was both good to mount the device and keep it safe from theft, rain and curious children. The majority of investigators (10 out of 12) had no difficulties with the removal and re-insertion of the SD card within the device, and only four reported problems during data downloading which consisted mainly of technical and user-related card reader issues (3/4).

Asides from the device in the Fajara, Gambia site that showed a poor level of agreement between the period averages from its two sensors, every other device revealed an acceptable level of agreement between its sensors A and B (Table 4). The sensor in Fajara, Gambia has since been replaced by a new one in preparation for the one-year longitudinal PM_2.5_ measurement.

## 4. Discussion

We found that it is practical and feasible to use low-cost air quality sensors to produce a network of air pollution data across several sub-Saharan African countries. Overall, the study generated a high data recovery rate of regular measurements of airborne PM_2.5_ concentrations with a granularity of 1–2 min over one month; only one of the 13 sites was unable to collect data due to a technical problem. Our data recovery rate of 94% compares favorably with that achieved by a field evaluation of a similar device alongside eleven others carried out by Feenstra et al. [34] in CA, USA (96%). Feenstra and colleagues evaluated twelve different sensors [namely, Shinyei PM Evaluation Kit (Shinyei Tech., China), Alphasense OPC-N2 (Alphasense, Essex, UK), TSI AirAssure (TSI Inc., MN, USA), Hanvon N1 (Hanwang Tech. Co., China), Airboxlab Foobot (Airboxlab, Luxembourg), Kaiterra LaserEgg (Kaiterra Ltd., Crans-Montana, Switzerland), PurpleAir PA-II (PurpleAir, UT, USA), HabitatMap Air Beam (HabitatMap, Brooklyn, NY, USA), SainSmart Pure Morning P3 (SainSmart, Canada), IQAir Air Visual Pro (IQAir, Zurich, Switzerland), Uhoo (uHoo Ltd., Singapore) and Aeroqual AQY (aeroqual, Auckland, New Zealand)] and obtained data recovery ranging from 71–98%. It is worth noting that the Purple Air II, which is a similar type to the one we used, achieved data recovery of 96% which is higher than the 94% we obtained in our study. Mukherjee and colleagues during their study to examine the performance of two models of PM sensors (the AirBeam and the Alphasense Optical Particle Counter -OPC-N2) over a 12-week period in the Cuyama Valley of California, reported approximately 100% for hourly data for the AirBeam sensors and over 92% hourly data recovery for the OPC-N2 sensors. The cause of missing data for OPC-N2 sensors was reported to be communication issues related to the data logger rather than the sensor itself. With the data logger issue neglected, data recovery was approximately 100% for hourly data. Furthermore, we deduced that the availability of electricity could have mainly influenced the almost 100% data recovery by the AirBeam and OPC-N2. The lower percentage data recovery during our study was mainly due to power cuts [27,34].

One of the biggest challenges experienced across the sites came from power outages due to irregular power supply. We had anticipated this based on our experience in exposure assessment in SSA and as such we made provision for one power bank per site. Some data were, however, still lost during the switch over to the power banks during power outages. In some cases where power outages occurred at night and/or where the power banks run out of charge at a time the exposure scientist was not available, data loss occurred. Some sites improvised by purchasing an additional power bank. Desouza and colleagues, during their study that highlighted how a sensor network through citizen science efforts can be a valuable way of increasing awareness about air pollution in communities, reported loss of power at some schools which subsequently affected their data recovery [35]. The study by Desouza et al., however, demonstrated how a citizen science approach and low-cost monitors can assist citizens in the various communities to understand the role they can play in improving air quality and to ask more of their local policymakers and government. West and colleagues also reported loss of data due to power outages during the air pollution measurements using the Dylos Air Quality Monitors (Dylos Corporation, Riverside, CA, USA) [36]. Another challenge they reported was that Dylos devices only had a 6 h battery life so concentrations during certain periods were not measured which might lead to misrepresentation of air pollution in that location. It is therefore important for future studies to understand the situation of the electricity supply at a location and explore multiple ways of addressing this and any challenges at the specific location before deployment. Understanding the electricity situation at a location could also provide guidance for choosing the suitable device to be used and also whether an external power source is required (power banks or small portable solar panels). Okello and colleagues published a practical document and checklist that can be used at an initial project planning stage for air pollution studies [37].

Under ideal conditions with availability of adequate resources, exposure scientists generally tend to use >22 h as acceptable cut-off from 24 h day data, thus taking ≤10% data loss as being manageable. Ultimately, how much data loss is acceptable from low-cost sensor networks needs to be clearly defined based on the study or project design. In order for a certain amount of data loss to be accepted, the remaining data must be useful for the purpose for which it was collected. If the sensor network data shows patterns which seem plausible based on other studies, or which on probing have an explanation, then the data themselves can be accepted because they have revealed something useful. Much as it would be ideal to have over 85% data recovery [34,35], shorter data recoveries (for example 50% which translates to 12 h a day) at local level could also stimulate new discussions about air pollution within the community through citizen science efforts and help identify potential air pollution “hot spots”. This can be a valuable way of spreading awareness and having public discussions, provided the potential deficiencies in the data are also part of the conversation. For example, pattern of peaks in data in the communities can provide an indication of the major sources of pollution. These patterns can be correlated with local activities in the community which could include transportation, rubbish burning and cooking within the communities [35].

The second important practical issue encountered was related to identifying a suitable location, particularly given the need for regular and safe access to the device to remove and re-insert the SD card to acquire stored data. Other device setup issues included extra costs for setup, e.g., paying a technician to set up the device, buying extra power banks, electricity cables, and card readers. Sites where devices were connected to Wi-Fi experienced some problems in initially connecting the device to wi-fi and during the extraction of data from the PurpleAir server via the website. All of these practical and local problems were solved through a project WhatsApp^®^ (Facebook, Menlo park, CA, USA) group or though one-on-one online remote discussions between the specific site coordinators and the principal investigator. It is important to note that despite many pre-deployment concerns relating to the safety of devices (i.e., from theft and tampering), no devices were stolen or tampered with. Discussions with people in the community at each site are likely to have helped in this regard and ensured that the local community appreciated the importance of the devices and the project. Furthermore, devices were deployed in a secure location at a height approximately 1.5 to 2 m above the ground at each location.

Our study demonstrates that it is practicable and feasible to establish a network of low-cost devices to provide real-time data on local PM_2.5_ concentrations in SSA countries simultaneously. The network and the resulting data could be utilized to raise public, societal and policymaker awareness about air pollution. Citizen science and low-cost sensor approaches have been utilized in a number of recent studies to increase awareness about ambient air pollution in targeted communities in SSA [35,36]. Through workshops, the researchers raised awareness of the issue of air pollution and brought together all stakeholders to discuss air pollution issues in all the locations where the studies have taken place.

There has been a gradual increase in efforts to measure ambient air pollution over the last decade [35,38,39] although most of the air quality measurement efforts in LMICs have been focused on household air pollution [6,40,41]. The lack of ambient air pollution data across SSA is likely to constrain development of societal awareness of the problems around poor air quality. There is a need for good quality, freely available real-time information on air pollution in SSA settings in order to generate the necessary interest in reducing emissions and improving air quality.

Efforts to reduce air pollution in high-income countries (HICs) have tended to be driven by public opinion, pressure groups and advocacy. Mortality and morbidity attributable to air pollution have, however, not decreased on a global level and there is a need for societal and structural changes in LMICs to tackle the problems of poor air quality [42]. The Lancet commission on air pollution recently stressed that nearly 92% of pollution-related deaths occurred in LMICs [2,43]. Given that this figure is almost entirely based on data obtained from LMICs outside Africa, the magnitude of the risk attributable to ambient air pollution has not been well documented for the African continent. Seeking effective and affordable ways to quantify the magnitude of ambient air pollution is key to increasing awareness and generating policy level change.

Although we did not have reference monitors at each of the MA3 sites, all the Purple Air-II-SD devices were factory calibrated. Purple Air II device PM_2.5_ measurements have shown high level of agreement with reference air quality monitors. Feenstra and colleagues reported high correlations of *R*^2^ > 0.96 and *R*^2^ > 0.90 for 24 h means between Purple Air PM_2.5_ mass measurements with co-located FEM GRIMM (Federal Equivalent Method GRIMM) and PM_2.5_ mass measurements co-located with FEM BAM (Federal Equivalent Method Beta Attenuation Monitors) PM_2.5_ data, respectively, for an 8-week deployment [35]. Our analysis of internal validity of each Purple Air revealed a high level of agreement between the two sensors (A and B) except for one device in The Gambia (Fajara). It is also worth noting the four sites in Enugu, namely, New Haven, Abakaliki Road, Trans-Ekulu and Goshen had a similar level of PM_2.5_ average concentrations of 33.0 μg/m^3^, 28.8 μg/m^3^, 30.3 and 30.3 μg/m^3^, respectively.

The time stamp on the in-built Real-Time Clock (RTC) within the Purple Air devices used experiences a lag or delay which is proportionate to the amount of time it stays without being connected to the internet. This lag or delay is known as the time drift. Starting from its time on the manufacturer’s shelf before procurement till when it was eventually installed, this time drift can be in minutes or days. When the device is connected to the internet, the RTC connects to an internet clock and updates its timestamp [3,30]. The two study sites that were permanently with Wi-Fi had little or no time shift. The other sites that were not connected to the internet had time drifts as much as 10 min over the period between purchase and the end of data acquisition. At locations where continuous internet connectivity is not available, connecting the Purple Air sensor to the internet a few minutes a week will help to update the RTC time. A mobile phone hotspot can serve this purpose when a wireless internet system is unavailable [3].

## 5. Conclusions

Concerted efforts need to be put in place to advocate for cleaner air in communities in SSA. Ambient air pollution quantification needs to be widespread so that the actual level of pollution at each location is mapped out and documented. Using this information as an advocacy tool, there is then a need to engage both the citizens and the policymakers on the issue of formulation and enforcement of legislation promoting cleaner air. This will include, but not be restricted to, banning of importation of old or poorly maintained petrol or diesel engine vehicles, indiscriminate burning of refuse, smoking in public places and use of dirty fuels for cooking [3]. Providing access to real-time data and training on what information means will enable primary health clinicians help their patients with respiratory diseases and multimorbidity better understand, manage and avoid potential triggers to symptoms within their daily lives. The information is also likely to assist in self-management of patients with respiratory diseases.

This study was not without weaknesses. There were insufficient funds and logistic strength to cover all countries in sub-Saharan Africa. Moreover, the choice of the study site was opportunistic and was determined by the country and town that the study participants were from. This led to a country having multiple sites (Nigeria) while many other countries were not represented at all. It would be informative and ideal to be able to cover most, if not all, sub-Saharan African countries where ambient air pollution concentrations are currently unrecorded. Power-related issues were a major practical challenge experienced by over a third of the investigators in our study, a finding that is not unusual in SSA countries [3,44]. With the benefit of hindsight, each Purple Air device should be accompanied by at least two Anker power banks to ensure the device is continuously powered.

Based on the limitations above, we recommend: future longitudinal studies that aim to record ambient air pollution in SSA should assess the electricity situation at each location and explore ways of how this can be addressed; have a coordination plan between the sites and the principal investigator (or person with experience using the devices), in addition to user guides so that the process of troubleshooting is fast. Future studies should also involve communities prior to deployment of devices as this foster’s community “buy-in” and advances in community involvement [3]. A follow-up MA3 study, to run over one calendar year (February 2020 to January 2021) is currently ongoing to enable us to execute spatial-temporal analysis and reveal the effects of seasonality on air pollution in the locations taking part. A policy level advocacy initiative has also been incorporated into this next phase of the work.

## Figures and Tables

**Table 1:** Characteristics of the 13 Sites Participating in the MA3 Pilot Study (July 2019).

Country	Town & City	Town Description	Season	Device Mounting Place	Wi-Fi Connection	Data Download Method
Benin Republic	Akpakpa, Cotonou	Urban	Wet	Hospital premises	No	SD card manually
Burkina Faso	Balkuy, Ouagadougou	Urban	Wet	Residential premises	No	SD card manually
Cameroon	Douala, Douala	Urban	Wet	Hospital premises	No	SD card manually
The Gambia	Fajara, Kombo	Urban	Wet	Residential premises	Yes	PurpleAir Website *
Kenya	Ngong Road, Nairobi	Urban	Wet	Residential premises	Yes	PurpleAir Website *
Nigeria	Bariga, Lagos	Urban	Wet	Hospital premises	No	SD card manually
Nigeria	New Haven, Enugu	Urban	Wet	Residential premises	No	SD card manually
Nigeria	Abakaliki Rd, Enugu	Semi-Urban	Wet	Residential premises	No	SD card manually
Nigeria	Trans-Ekulu, Enugu	Urban	Wet	Residential premises	No	SD card manually
Nigeria	Goshen, Enugu	Urban	Wet	Residential premises	No	SD card manually
Nigeria	Nnewi, Anambra	Urban	Wet	Residential premises	No	SD card manually
Nigeria	Awka, Anambra	Urban	Wet	Residential premises	No	SD card manually
Uganda	Ntinda, Kampala	Urban	Wet	Office premises	No	SD card manually

* Automatically uploaded to the Purple Air website when device is connected to Wi-Fi.

**Table 2:** Data Recovery Rates and Frequency Distribution of PM_2.5_ Measurements by Three Different Threshold Categories by PA Time Periods in the MA3 Pilot Study.

Country *	Town & City	Number of Records Logged (*n*)	PA° Time Periods (*N*)	Data Recovery Rates (%)	>10 μg/m^3^ n > 10 (%)	>25 μg/m^3^ n > 25 (%)	>250 μg/m^3^ n > 250 (%)
The Gambia	Fajara, Kombo	20,636	22,320	94.7%	11,455 (55.5%)	1644 (8.0%)	78 (0.4%)
Burkina Faso	Balkuy, Ouagadougou	21,142	22,320	94.7%	16,026 (75.8%)	4647 (21.9%)	<0.1 (0%)
Benin Republic	Akpakpa, Cotonou	30,799	33,480	92.0%	29,262 (95.0%)	9178 (29.8%)	3 (0.01%)
Nigeria	Abakaliki Rd, Enugu	32,999	33,480	98.6%	30,437 (92.2%)	15,972 (48.4%)	13 (0.04%)
Nigeria	Trans-Ekulu, Enugu	31,139	33,480	93.0%	28,428 (91.3%)	15,178 (48.7%)	28 (0.09%)
Nigeria	Goshen, Enugu	35,322	33,480	105.5%	32,512 (92.0%)	18,084 (51.2%)	4 (0.01%)
Nigeria	New Haven, Enugu	31,241	33,480	93.3%	29,569 (94.6%)	18,811 (60.2%)	4 (0.01%)
Nigeria	Awka, Anambra	31,500	33,480	94.1%	29,343 (93.2%)	20,003 (63.5%)	18 (0.06%)
Kenya	Ngong Rd., Nairobi	22,320	22,320	100.0%	21,322(95.5%)	16,944 (76.0%)	11 (0.05%)
Nigeria	Nnewi, Anambra	21,078	22,320	94.4%	20,500 (92.3%)	16,944 (80.4%)	173 (0.82%)
Uganda	Ntinda, Kampala	21,312	22,320	95.5%	21,293 (99.9%)	19,605 (92.0%)	276 (1.3%)
Nigeria	Bariga, Lagos	24,148	33,480	72.1%	24,062 (99.6%)	23,113 (95.7%)	22 (0.09%)

°PA stands for PurpleAir time periods. This represents the ideal number of logs each device is meant to have over the 31 days, assuming it logs every 80 s (33,480) or 120 s (22,320). * Cameroun was excluded from the analysis since the devices did not log any data.

**Table 3:** Practical Challenges Identified during Field Use of PurpleAir-II-SD AQM Sensor (*N* = 12).

Issues	Specific Characteristics	Reports *n* (%)
**Power Issues**	- No power problems reported	5 (41.7%)
	- Irregular electricity supply	4 (33.3%)
	- Additional power bank needed	1 (8.3%)
	- Use of electricity generators	2 (16.7%)
**Device Setup**	- No setup issues reported	6 (50%)
	- Finding suitable location for device setup	2 (16.7%)
	- Incurring extra cost for assisted device setup	2 (16.7%)
	- Keeping device safe from theft, children, etc.	1 (8.3%)
	- Connecting to Wi-Fi	1 (8.3%)
**Memory Card**	- No SD memory card problems	10 (83.3%)
	- Problems with removal and re-insertion of SD card	2 (16.7%)
**Data Download**	- No data downloaded problems reported	8 (66.7%)
	- Extracting data from Wi-Fi	1 (8.3%)
	- Card reader issues	3 (25%)

**Table 4:** Level of Internal Agreement between Purple Air Sensors A and B at Each MA3 Site.

Country	Site/Town	Area	Period Average PM_2.5_ (μg/m^3^) A	Period Average PM_2.5_ (μg/m^3^) B
Burkina Faso	Ouagadougou	Balkuy	19.3	20.2
Gambia	Fajara	Kombo	15.6	0.5
Cameroon	Douala	Douala	-	-
Nigeria	Enugu	New Haven	33.0	30.4
Nigeria	Anambra	Nnewi	52.3	52.7
Nigeria	Anambra	Awka	33.4	33.2
Kenya	Nairobi	Gong Road	38.8	36.2
Uganda	Kampala	Ntinda	91.1	87.8
Benin	Cotonou	Akpakpa	22.1	22.9
Nigeria	Enugu	Abakaliki Rd.	28.8	29.2
Nigeria	Enugu	Trans-Ekulu	30.3	29.0
Nigeria	Enugu	Goshen	30.3	29.9
Nigeria	Lagos	Bariga	56.3	57.1

## Supplementary Materials

The following are available online at https://www.mdpi.com/1660-4601/17/19/7243/s1, S1: Map showing the gap in the knowledge of Ambient Air Quality index in sub-Saharan Africa with an accompanying US Environment Protection Agency (USEPA) Air Quality Index table; S2: Performance of the GRIMM reference method versus Purple Air Sensor (named unit 8464 in the field evaluation); S3: Measuring Air Quality for Advocacy in Africa (MA3): Protocol for baseline feasibility and practicality study; S4: Protocol for Purple-Air-II-SD Time stamp Shift in the MA3 study.
