# Supplementary material for: Measuring Air Quality for Advocacy in Africa (MA3): Feasibility and Practicality of Longitudinal Ambient PM2.5 Measurement Using Low-Cost Sensors
Source: Int J Environ Res Public Health. 2020 Oct 3;17(19):7243. doi: 10.3390/ijerph17197243 (PMC7579047; doi:10.3390/ijerph17197243)
Supplement: Supplementary file 1 [file ijerph-17-07243-s001.pdf]

Measuring Air Quality for Advocacy in Africa (MA3):

Feasibility and practicality of longitudinal ambient $PM_{2.5}$ measurement using low-cost sensors

Authors:
Babatunde I. Awokola[1,2,3* #], Gabriel Okello [4 #] (Joint first Authors), Kevin Mortimer[2,5], Christopher P. Jewell[1], Annette Erhart[6], Sean Semple[7]

*Corresponding Author, # Joint first author

SUPPLEMENTARY FILES

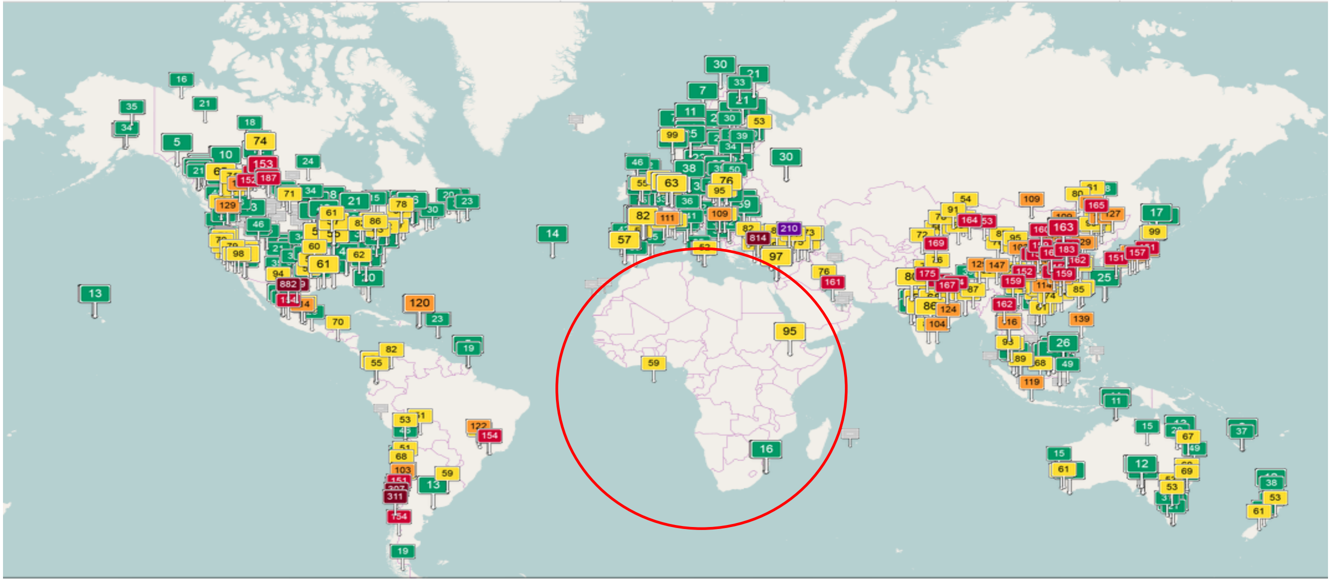

$PM_{2.5}$ µg /m^3	AQI	Advice
0-12	Good	Air quality is satisfactory and poses little or no health risk.
12.1-35.4	Moderate	People who are unusually sensitive to particle pollution may experience respiratory symptoms.
35.5-55.4	Unhealthy for sensitive groups	Heart/lung disease and elderly/ children advised reducing prolonged exertion
55.5-150.4	Unhealthy	Heart/lung disease/elderly/children avoid prolonged exertion; everyone reduces prolonged exertion
150.5-250.4	Very unhealthy	Heart/lung disease avoid all physical activity; everyone avoids prolonged exertion
250.5+	Hazardous	Heart/lung disease remain indoors; everyone avoids physical activity

Supplementary File 1 (S1) : Map showing the gap in the knowledge of ambient air quality index in sub-Saharan Africa with an accompanying United States Environmental Protection Agency (USEPA) Air Quality Index ***Source:*** www.onairschools.org/2017/09/07/air-quality-real-time-map

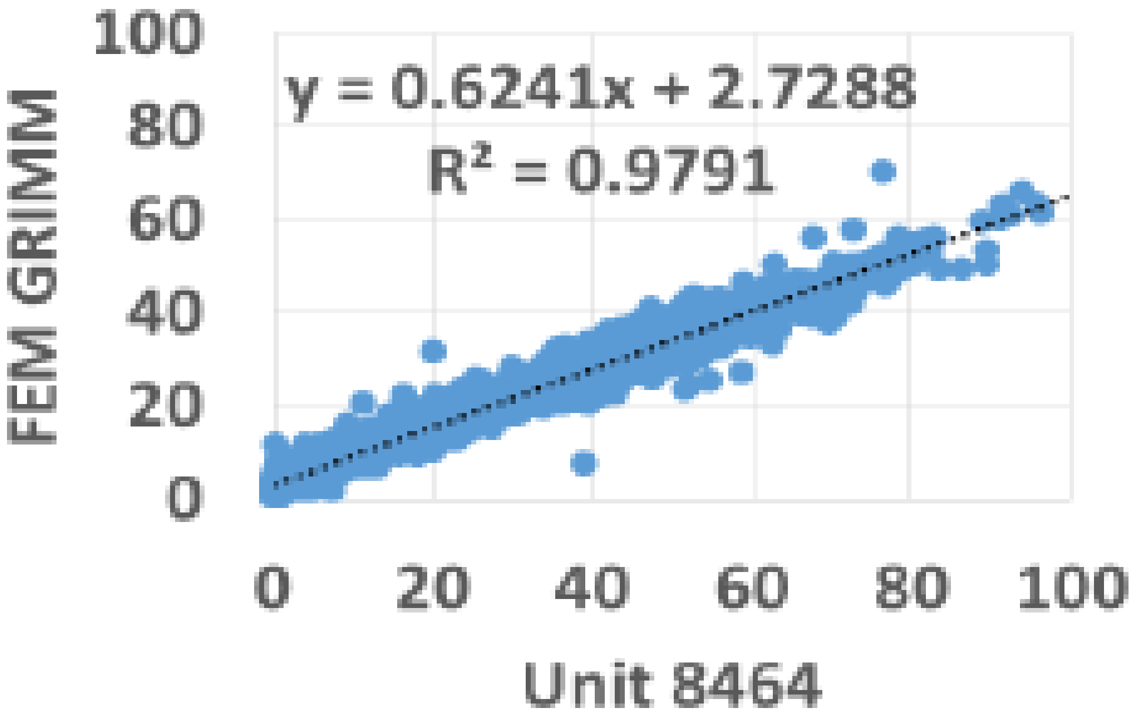

Supplementary File 2 (S2): Performance of the GRIMM reference method *versus* Purple Air sensor (named unit 8464 in the field evaluation). FEM=Federal Equivalent Method. $PM_{2.5}$ five-minute mean, measured in μg/m^3.

Source: http://www.aqmd.gov/docs/default-source/aq-spec/field-evaluations/purple-air-pa-ii---field-evaluation.pdf?sfvrsn=4 (Page 7)

Supplemental file 3 (S3)

MEASURING AIR QUALITY FOR ADVOCACY IN AFRICA- MA3

PROTOCOL FOR BASELINE FEASIBILITY & PRACTICALITY STUDY

Version	1.0
Acronym	MA3 Protocol
Sponsor	African Centre for Clean Air (ACCA)
Funders	Liverpool School of Tropical Medicine (LSTM)
Principal Investigators	Babatunde Awokola MBBS, FWACP, ATSF
	Gabriel Okello PhD
	Sean Semple PhD
	Kevin Mortimer PhD
Co-Investigators	PATS MECOR Air Quality Measurement Trainees 2019 Cohort
	Herve Lawin PhD
	Bertrand Ngahane
Study Statistician	Chris Jewell PhD
Study Co-ordinating Centre	ACCA
Authors	Babatunde Awokola, Gabriel Okello, Chris Jewell, Kevin Mortimer, Sean Semple

Summary

What is the key question?
Is it practical and feasible to gather data on air quality using low-cost particle sensors deployed across sub-Saharan Africa?
**What is the bottom line?**
Air pollution is a big global health problem and low-and-middle-income countries, where the use of biomass fuel is prevalent. Ambient air pollution, especially in urban areas in Africa, is largely as a result of emissions from old and poorly maintained vehicles, high sulphur fuels, dust and fumes from industries, smoking and roadside rubbish burning in addition to biomass fuels.
**Why read on?**
Air pollution by particulate matter (PM) have documented health effects: COPD, asthma, lung cancer, heart disease etc. We need to quantify the extent of the pollution as a tool for advocacy for cleaner air.

Title: Measuring Air Quality for Advocacy in Africa: Longitudinal measurement of Ambient $PM_{2.5}$ in Sub Saharan African countries

Research Question:

Is it practical and feasible to gather data on air quality using low-cost particle sensors deployed across sub-Saharan Africa?

Objective:

Overall objective is to execute an air quality measurement primary study with a large geographical coverage utilising low-cost particle sensors and reliable longitudinal designs with a view to generating ambient $PM_{2.5}$ data that is truly representative quantitative measurements of ground- level air pollution in sub-Saharan Africa.

Specific objectives are:

1. To assess the ambient $PM_{2.5}$ pollutant level over a four-week period in the nine sub-Saharan African countries (The Gambia, Tanzania, Kenya, Uganda, Nigeria, Burkina Faso, Sudan, Cameroon & Benin Republic) using a low-cost air quality measurement device (Purple air II SD device)
2. To compare the ambient $PM_{2.5}$ pollutant levels in each country with the WHO air quality standard threshold for ambient $PM_{2.5}$

Rationale/Background

Exposure to ambient air pollution is increasing becoming a significant health-related environmental issue. It is estimated that 3 billion people, *i.e.* 40% of the world's population, are exposed daily to air pollution with a majority living in low-and-middle-income-countries (LMICs). Air pollution originates largely from incompletely combusted solid fuels used in households. In urban areas in Africa, ambient air pollution also arises from emissions from old and poorly maintained vehicles, high sulphur fuels, dust and fumes from industries, smoking and roadside rubbish burning. The overall morbidity related to air pollution is further increased by its independent effect on obstructive lung diseases.

Why measure ambient air pollutants?

Inhalable, thoracic and respirable fractions of particulate matter (PM) have documented health effects associated with them. These health effects are determined to a large extent by the concentration of particles, surface area of particles, chemical constituents of particles and the biological activity of the particles. Some of these health effects are COPD, asthma, lung cancer, heart disease, stroke, arterial thrombosis, hypertension to mention a few. Measuring ambient $PM_{2.5}$ will help identify the main sources and quantify the extent of the pollution with the aim of enacting advocacy, which will eventually lead to the establishment and reinforcement of

strict air quality regulation in the sub Saharan African region. These data will assist in supporting African governments to implement evidence-based policies and programmes and could also influence behavioural change in some communities/countries i.e some countries can copy and replicate successful programmes from others.

Study Design

The study is a four-week multi-centric longitudinal air pollution monitoring to evaluate the air quality in nine countries across Africa using low cost sensors. Data on ambient $PM_{2.5}$ will be collected continuously.

Participants

The Physicians participating in the African Centre for Clean Air (ACCA) facilitated air quality monitoring training will carry out the $PM_{2.5}$ measurement in the following locations:

1. Fajara, The Gambia
2. Lagos, South-Western Nigeria
3. Nnewi, Eastern Nigeria
4. Awka, Eastern Nigeria
5. Enugu, Eastern Nigeria
6. Kampala, Uganda
7. Ouagadougou, Burkina Faso
8. Nairobi, Kenya
9. Mbeya, Tanzania
10. Khartoum, Sudan
11. Douala, Cameroon
12. Cotonou, Benin

Sampling Strategy

Sampling frame

Ground level ambient air at a hospital, clinic or university setting within the general metropolitan area of each city. The probable locations are listed above under participants

Sampling item

Ambient Particulate matter, 2.5 mm ($PM_{2.5}$)

Sampling Tool

Purple Air II SD sampling sensor

- ✓ Primary output variable is $PM_{2.5}$ concentration (μg/m^3)
- ✓ Records data every 80 seconds
- ✓ Also records
    - o Temperature
    - o Humidity
    - o Wifi signal strength and
    - o Uptime
- ✓ Records particle number concentrations in various size bins (0.3, 1.0, 2.5, 5, 10 mm)
- ✓ It has two methods of operation:
    - Uploads in real-time to server via wifi (or mobile phone hotspot)
    - Logs to SD card – so data always recoverable if wifi signal lost
- ✓ The device has two identical sensors and so provides the facility for internal validation.

Sampling Sequence

PLEASE USE THE POWER PACK GIVEN FOR POWERING DEVICE AND THE SD CARD FOR STORING DATA.IF THERE IS ACCESS TO UNINTERRUPTED POWER AND WIFI, GET NECESSARY PERMISSION AND USE THIS.

1. Set up the sampling tool-Purple air SD sampling sensor. Installation tips are:

    i. The housing is designed to protect the device from elements while allowing air to flow freely past the two laser counters
    ii. The power supply should be mounted so that it will not be submersed in water or affected by rainfall
    iii. Use a '**drip loop**' to prevent water from running down the wires and into the electronics
    iv. If possible, mount the sensor in a shady spot out of direct sun
    v. If possible, away from air conditioning vents and other local sources of pollution like barbeque coal grills etc.
    vi. Use either **cable ties** or **screws** to mount the sensor and power supply. Where reliable mains electric power is available, this should be used. In settings where there are frequent power outages occurring, power banks should be used to operate the device.
    vii. Connect the power cables to a power supply point and tuck the wires away.

2. Connect the sampling tool-Purple air SD sampling sensor to a power source.
3. Securely anchor/mount it in a safe location protected from theft and destructive elements of nature. Fasten using either cable ties or screws.
4. Connect Purple air to a wifi if available. To connect to a wifi network:
    a. Open a browser with the page http://www.purpleair.com/configure
    b. Next, disconnect your laptop or pc from your office/home wifi. The easiest way to do this is to switch the wifi off for next step.
    c. Connect to 'Air Monitor_XXXX' via your laptop wifi. XXXX is unique to each specific purple air device.

    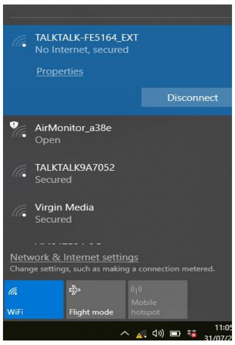

    d. Once you re-connect to the wifi, you might see messages such 'No internet, open' or 'Unable to connect to internet'. Do not worry about this.
    e. Return to the browser and the 'configure' page
    f. Press 'connect to sensor'
    g. Configure wifi by choosing your wifi network from the list of options, entering the password (if applicable) and then pressing save
5. If wifi is unavailable or not dependable, utilize the SD card component of the measuring equipment. It is however important to try to connect to wifi (or mobile hotspot) at least every 7 days to let the real time clock re-establish a link to 'internet time'.
6. Zip and send the $PM_{2.5}$ longitudinal data to bawokola@gmail.com (copy gabrielokello@gmail.com every 7 days (weekly). This is particularly important for those capturing data using the SD card component of the Purple air device. ***
7. Ensure you do a daily check on the visibility of your sensor real-time via Purple Air website. This however applies if you are permanently connected to wifi.

8. While doing item 7, Carry out a quick check on the uptime graph (sensor tab). This should be a straight line. Note that this only shows activity for the previous 8 hours.
9. Download the primary data file for that day using the online downloader.
10. Check that the downloaded data file has no significant data gaps. There should be 1080 records per 24h period. If <972 (90%), then we recommend downloading via manual process (i.e. SD data card) to see if missing data are due to poor wifi connection
11. Check that the data 'makes sense' and that sensor A and sensor B are in broad agreement on the one-hour average tab. (R2>0.90) and record this.
12. Save final file for that day and record average 24h $PM_{2.5}$ value in separate database

Variables

Outcome variable: The primary outcome variable is the percentage of $PM_{2.5}$ data logged by each device given the total number of minutes in the month of July 2019. Secondary outcome variables are challenges experienced during the installation, use, maintenance and data download from the Purple Air -II-SD devices used.

Exposure variables : Sampling site (urban/semi-urban city in sub-Saharan Africa) ; Meteorological conditions: dry and rainy season, road dust (unpaved roads, living on the road side, road capacity), traffic (high traffic area, traffic flow, fuel type, age of cars) , sources of domestic energy (coal/charcoal and other biomass fuel), solid waste combustion, bush burning, commercial activities (food preparation, street vendor, vehicular/motorbike taxi driver) , tree cutting and construction activities .

Data Collection: Baseline data about the sampling site will be collected using the questionnaire in Appendix 1. Install GPS essentials® app from the google play store or the apple app store onto your smart phone. Stand beside where the PurpleAir sensor is set up, activate the app and click on satellite. Wait for the app to acquire the GPS of your location, then record it on your form. Scan the fully filled form and send it to bawokola@gmail.com and copy gabrielokello@gmail.com. $PM_{2.5}$ concentration will be sampled as already highlighted under the sampling sequence section.

Statistical Analysis Plan

Longitudinal data from all the ACCA data measurement sites will be collated, checked for consistency and completeness, and cleaned. Trends and associations in the data will be investigated using longitudinal data methods (autoregressive timeseries models, hierarchical modelling for timeseries data). We will then calculate the probability that our $PM_{2.5}$ measurements exceed the current WHO standard for ambient $PM_{2.5}$, with the aim of generating data that quantifies the magnitude of air pollution in these sub Saharan African countries.

Timeline

The various phases of the research project are as in the Gantt chart below:

	June 7-16	June 17-30	July 1- 30	August 1-7	August 7-21	August 22-30	Sept. 1-10
**AQM Training**	■						
**Set up of PurpleAir at home countries**		■					
**Four weeks of data capturing**			■				
**Data upload**			■	■			
**Final Data upload**				■			
**Data Analysis**				■	■		
**Write-up**						■	■

Budget

Funds for this project comes from the research fund of Dr Babatunde Awokola, MRes/PhD student with the Department of Clinical Sciences, Liverpool School of Tropical Medicine (LSTM). This institutional slot for the PhD was made possible by the Medical Research Council Doctoral Training program, Lancaster University, LSTM and the Aldama Foundation. Details of the budget for this project is available on demand from the IMPALA/LuLi administrator Debbie Jenkins debbie.jenkins@lstmed.ac.uk

Strengths & Weaknesses

The strength of the study lies in the use of a low cost, high performing tool for ambient air pollution measurement. Furthermore, the potential for $PM_{2.5}$ to be measured and recorded in real-time is another strong point to allude to.

The downside to the project is the length of data collection. The period of one month is too short to show time series, execute spatial-temporal analysis and reveal effects of seasonality on air pollution.

Next phase

Leveraging on our one-month experience, we will progress into a one-year (12-calender months) measurement. This will generate the largest published dataset on ambient $PM_{2.5}$ globally. This, in turn, will serve as an advocacy tool for enacting laws that will lead to cleaner air for citizens of developing countries like in sub-Saharan Africa.

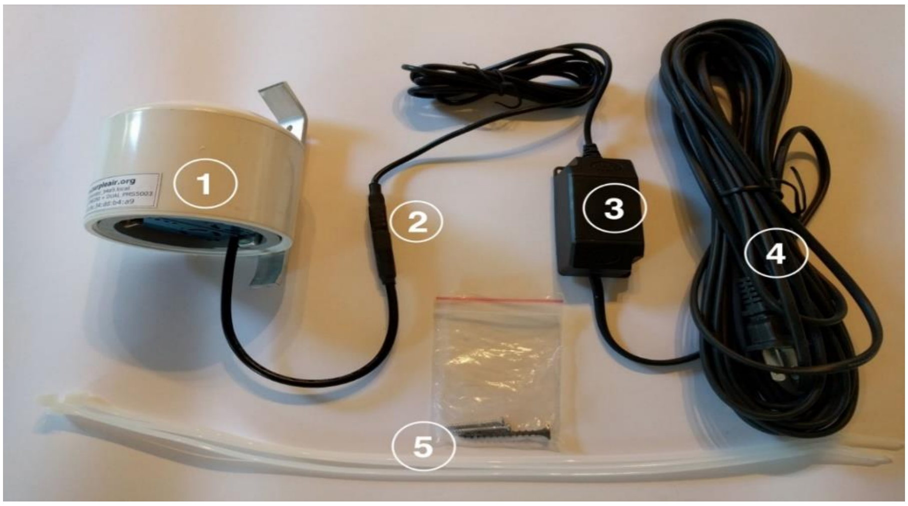

Figure 1: PurpleAir in the box

The labelled parts are as below:

1. Purple air II dual laser sensor
2. Micro USB connector
3. 5V 2A USB outdoor power supply
4. 17-foot power cable
5. Zip ties for mounting

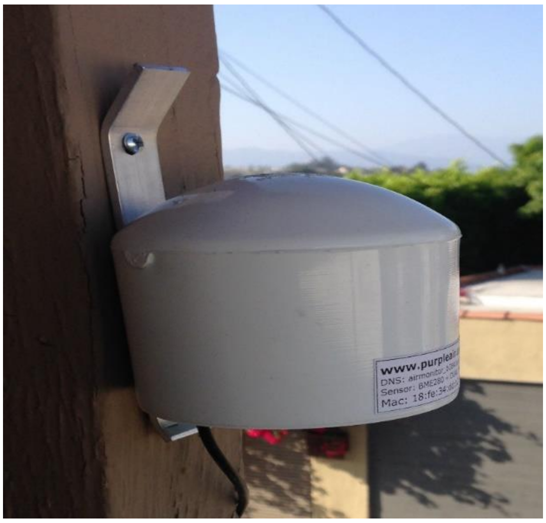

A

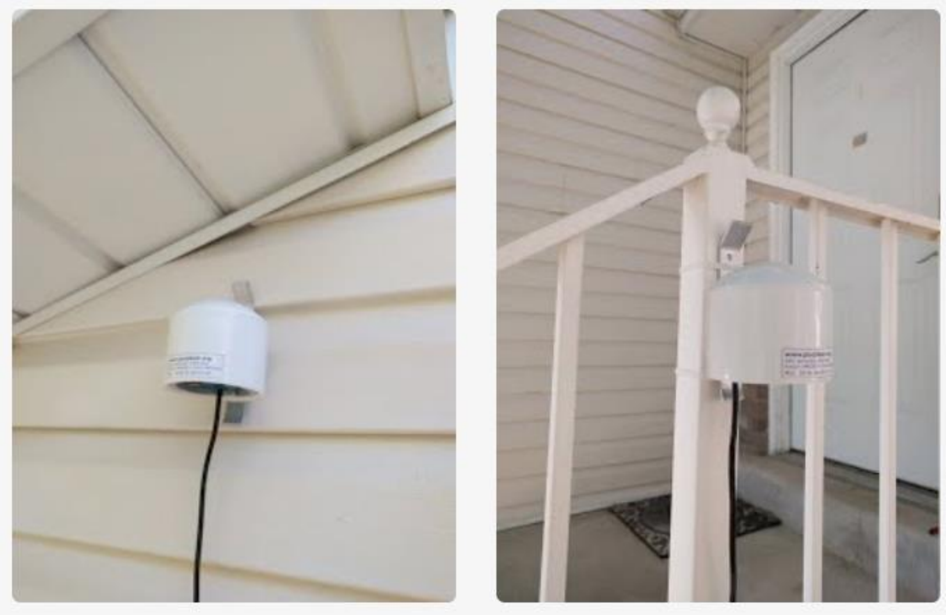

B C

Figures 2 A, B & C: Mounting the PurpleAir on the wall (A), under a shed (B) and on the stair handles (C).

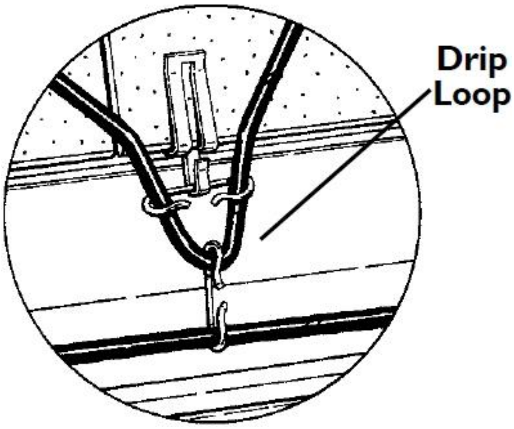

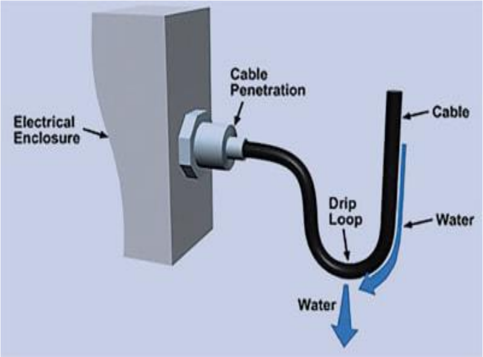

Figure 3: Drip loop to allow water to run off wires

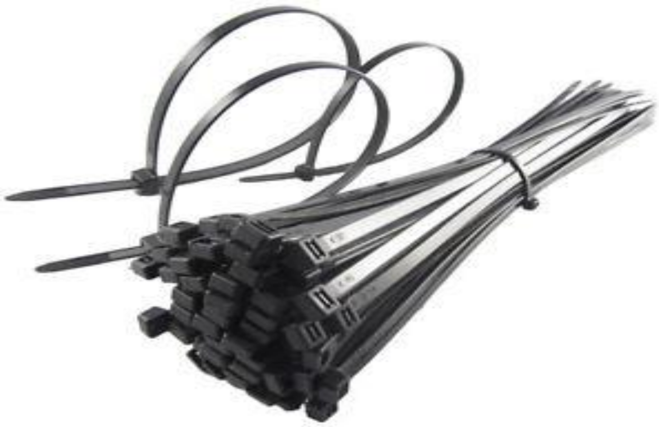

Figure 4: Black coloured ‘cable-tie’ for mounting the PurpleAir

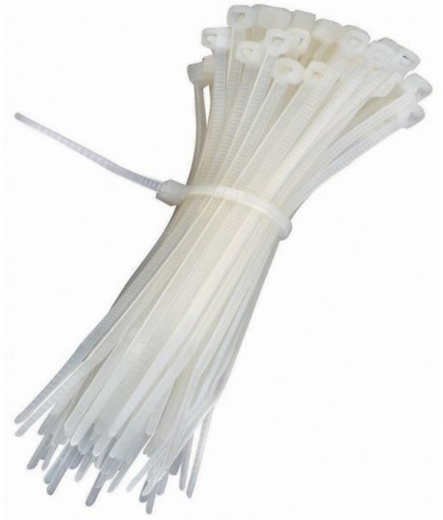

Figure 5: White coloured ‘cable-tie’ for mounting the PurpleAir

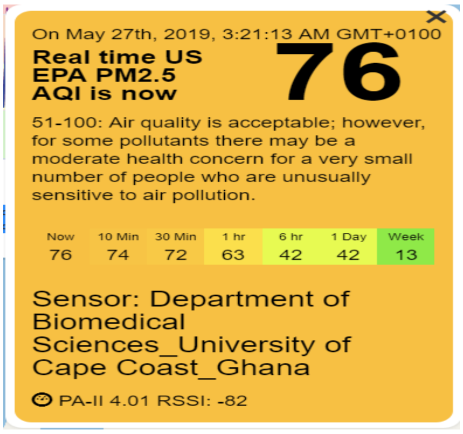

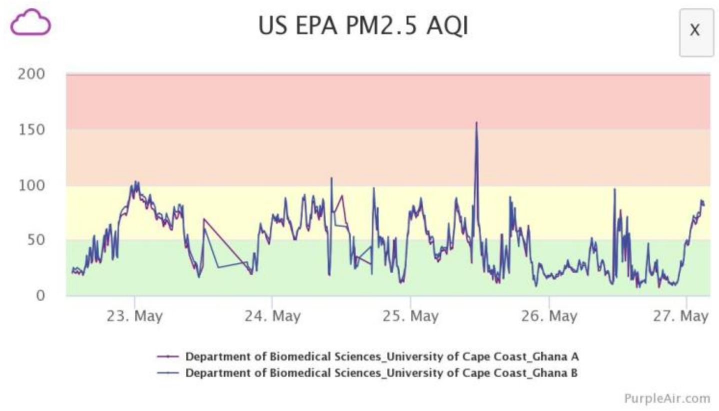

Figure 6: Downloaded sensor dashboard and graph from the PurpleAir maps website

Equipment Maintenance
Technical details

Purple air dual laser air quality sensor uses laser beams to detect the particles going past by their reflectivity, like dust shimmering in a sunbeam. The PM 2.5 and PM 10 micro-gram weights are calculated from the counts. The values are averaged every 20 seconds and graphically displayed on the purple air website.
PurpleAir displays the particulate matter numbers, in both the PM 2.5 and PM 10 range. It also tracks particle counts in six sizes between 0.3um and 10.0um in diameter.
PurpleAir sensors read very similar numbers to other sensors of the same type (laser counters). Some sensors use very different methods to measure particles and so we expect some disagreement with their numbers. Currently, Purple Air is seeking to know the amount of disagreement between these tools.
Every care is taken to place PurpleAir monitors in locations that are representative of the air we breathe every day. They are placed in neighbourhoods, on the side of houses, a few feet above our heads. They use the homeowner's Wi-Fi and power. Local pollution like cigarette smoke, BBQ's, fireplaces and idling cars can cause spikes in the short-term graphs.
Quality techniques like an initial decay test and correlation testing with new and older monitors can be used. One of the issues that faces these devices, and others, is what is called "drift". This happens with optical particle sensors because of dust that may settle inside the device and may cause an offset or drift over time. A maintenance schedule is necessary with any measuring device, but ours are relatively easy to maintain and cheap to service.
PurpleAir sensors can also be used indoors.
The simplest way to view our data is the PurpleAir Map(http://www.purpleair.com/map). Data is also available on MesoWest's network and others. We frequently share raw data with researchers.

Technical Specifications
Power Supply
Outdoor power supply with 17-foot leads:
Output Voltage: 5 Volt (V), Output Current: 2 Amps (A)
General Specifications
DC Power Supply Requirements: Typ:5.0 Min:4.5 Max: 5.5 Volt (V)
Active Current: ≤200 Milliampere (mA), Peak Current: ≤600 Milliampere (mA)
Working Temperature Range: -20 ~ +60 °C (-4 ~ +140 °F)
Working Humidity Range: 0~99%
Physical Size 85 x 125 Millimeter (mm) (3.5 x 5 Inches)
BME280: Pressure / Temperature / Humidity
Operation range (full accuracy)
Pressure: 300…1100 hPa
Temperature: -40…85 °C
Humidity sensor response time (τ63%): 1s
Accuracy tolerance: ± 3 % relative humidity
Hysteresis: ≤ 2 % relative humidity"
Dual laser particle counters for fault tolerance:
Range of measurement: 0.3~1.0; 1.0~2.5; 2.5~10 Micrometer (µm)
Counting Efficiency: 50%@0.3µm 98%@>=0.5µm
Effective Range: 0~500 µg/m^3
Maximum Range: * ≥1000 µg/m^3
Resolution: 1 µg/m^3
Maximum Consistency Error: ±10%@100~500µg/m^3 ±10µg/m^3@0~100µg/m^3
Standard Volume: 0.1 Litre (L)
Single Response Time: ≤1 Second
Total Response Time: ≤10 Seconds
Wireless Specifications
WiFi 2.4GHz, supports WPA/WPA2
Wireless Standards: 802.11 b/g/n
Certificates: FCC/CE/TELEC/SRRC
Tx Power:
802.11 b: +20 dBm
802.11 g: +17 dBm
802.11 n: +14 dBm
Rx Sensitivity:
802.11 b: -91 dbm (11 Mbps)
802.11 g: -75 dbm (54 Mbps)
802.11 n: -72 dbm (MCS7)
Type of Antenna: External, IPEX Connector, Ceramic Chip
*** Optional SD Card Slot and Real Time Clock:**
Extremely accurate DS3231 real-time clock (RTC) incorporates a battery backup and maintains accurate time keeping when main power to the device is interrupted.
A built in microSD card slot takes microSD cards up to 64GB for storage that will never run out. PurpleAir sensors receive firmware updates over the air when connected to the Internet.

References

1. Purple Air II dual laser air quality sensor Available from https://www.purpleair.com/sensors.
2. Gordon SB, Bruce NG, Grigg J, Hibberd PL, Kurmi OP, Lam KH, Mortimer K et al. Respiratory risk from household air pollution in low and middle-income countries *Lancet Respir Med* 2014; 2: 823-60
3. Katoto PDMC, Byamungu L, Brand AS, Mokoya J, Strijdom H, Goswami N, De Boever P, Nawrot TS, Nemery B . Ambient air pollution and health in Sub-Saharan Africa: Current evidence, perspectives and call to action. *Environmental Research* 2019; 173: 174-188
4. Okello G, Devereux G, Semple S. Women and girls in resource poor settings experience much greater exposure to household air pollutants than men: Results from Uganda and Ethiopia. *Environment International* 2018; 119:429- 437.
5. Okello G, Mortimer K., Lawin H., Semple S. Quantifying exposure to respiratory hazards in sub-Saharan Africa: planning your study. *AJRM*. 2020.14:2

APPENDICES

Practical Challenges in the Installation, use, maintenance and data extrication from Purple Air II In SSA

The use of any piece of technology is usually froth with challenges. The ability to manage and overcome these challenges determines the success of projects involving the use of these tools on the long run.

As part of our data collection, we request that all Co-Principal investigators should kindly document every challenge experienced from the onset till the end using the form in appendix 2. This will enable everyone who will use PurpleAir II subsequently be better armed to combat any challenge that may constitute a hurdle on the way of efficient and effective air quality measurement.

Appendix 1

Questionnaire for data collection site

GPS Coordinates ………………………………………………………………..

1. Country: …………………………………………………………………….
2. State……………………………………………………………………….
3. City/Town/Village…………………………………………………………….
4. City/Town/Village description: a. Urban……………… b. Rural…………

   c. Semi-urban………..
5. Period of data collection: dd/mm/yyyy to dd/mm/yyyy
   ………………………………………………………………………………
6. Season in data site during data collection i.e. dry, wet, harmattan etc
   ………………………………………………………………………………
7. Any active source of pollutant close by e.g. grill, incinerator, high vehicular traffic, biomass fuel use, street food vendors etc (list as many as is present

Kindly list out the issues and challenges encountered while installing, using maintaining or extracting data from PurpleAir II SD sensor. Please, be as clear and as comprehensive as possible. Please, write in capital letters or type for easy readability/legibility.

Installation Challenges
Issues with Purple Air Use and Maintenance
Challenges with Data Extraction
Others

Supplementary file 4 (S4)

PROTOCOL FOR PURPLE AIR-II-SD DEVICE TIME STAMP SHIFT IN THE MEASURING AIR QUALITY FOR ADVOCACY IN AFRICA (MA3) STUDY

Checking time stamp on purple air device operating without connection wifi

Introduction

The emergence of low-cost air quality devices offers new opportunities to simultaneously gather spatial and temporal air quality data in near-real-time, as well as engage citizens in active environmental monitoring in sub-Saharan Africa (SSA). This subsequently provides more capabilities in the assessment of human exposure to air pollution and identifying the factors leading to pollution in the various areas in SSA.

Sensor platforms are currently available to monitor a range of air pollutants and new devices are continually being introduced (Piedrahita et al., 2014). We are therefore going through a paradigm shift in how and who is monitoring air quality (Lewis and Edwards, 2016). There has been introduction of devices that are relatively lower in cost, easier to use and less bulky than traditional equipment, and offer the prospect for citizens and communities to monitor their local air quality that may affect their health (Snyder et al., 2013).

Significance:

Data quality is a pertinent concern, especially in citizen science applications, where citizens are collecting and interpreting the data. Operating any piece of technology usually comes with some challenges. The capability to manage and overcome these challenges determines the success of projects involving the use of these tools in the long run.

Extremely accurate DS3231 real-time clock (RTC) in the Purple Air II SD incorporates a battery backup and maintains accurate time keeping when main power to the device is interrupted, however there’s a possibility of shifting of time stamps on devices that are operational but not connected to wifi.

Objective:

Overall objective is to check whether there's a shift in time (minutes lost) when the Purple Air-II-SD devices are operated without connection to wifi.

Sampling Tool

Purple Air II SD sampling sensor (check purple air website or MA^3 protocol for specifications)

Method for checking shift in time stamp on Purple Air II SD

13. Set up the Purple air II SD sampling sensor.
14. Connect the Purple air SD sampling sensor to a power source.
15. If device is already operational, switch off the sensor by disconnection from a power source at a specific time for 10 to 20 seconds and.
16. Record the time the disconnection was done.
17. Connect the device to power source again, measure for five more minutes, then download and send the file to bawokola@gmail.com and copy gabrielokello@gmail.com
18. For devices where time shift has occurred, will require logging on to the internet using wifi or phone hotspot for five minutes.

Procedure for connecting to wifi

Connect Purple air to a wifi if available. To connect to a wifi network:

a. Open a browser with the page
 http://www.purpleair.com/configure
b. Next, disconnect your laptop or pc from your office/home wifi. The easiest way to do this is to switch the wifi off for next step.
c. Connect to 'Air Monitor_XXXX' via your laptop wifi. XXXX is unique to each specific purple air device.

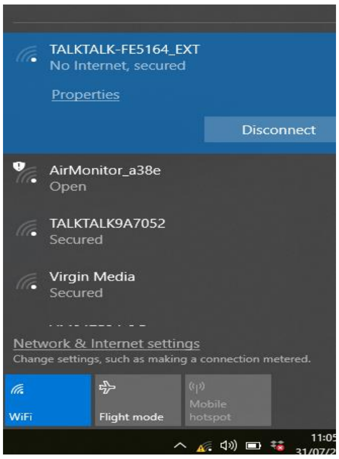

d. Once you re-connect to the wifi, you might see messages such 'No internet, open' or 'Unable to connect to internet'. Do not worry about this.
e. Return to the browser and the 'configure' page
f. Press 'connect to sensor'
g. Configure wifi by choosing your wifi network from the list of options, entering the password (if applicable) and then pressing save
h. The device will only be connected for five minutes and then disconnected.
i. Measurement will then continue without connection to wifi

NOTE: In case of any challenges, a step by step procedure will be carried out with Babatunde at a prior agreed and scheduled time.

References

1. Lewis A Edwards P. Validate personal air-pollution sensors. *Nature* 2016. 535 (7610), 29–31.
2. Piedrahita R, Xiang Y, Masson N, Ortega J, Collier A, Jiang Y, et al. The next generation of low-cost personal air quality sensors for quantitative exposure monitoring. *Atmos. Meas. Tech*. 2014 7, 3325–3336.
3. Snyder E, Watkins T, Solomon P, Thoma E., Williams R., Hagler G., Shelow D, Hindin D, Kilaru V, Preuss P.The changing paradigm of air pollution monitoring. *Environ. Sci. Technol*. 2013. 47, 11369–11377.
